# Translation of a Falls Prevention Behavioral Intervention for Family Caregiver Administration

**DOI:** 10.1093/geroni/igab046.1733

**Published:** 2021-12-17

**Authors:** Victoria Panzer, Veronica Smith, Dorothy Wakefield, Richard Fortinsky

**Affiliations:** 1 Brookside Research & Development, Freeland, Washington, United States; 2 Brookside Research & Development, Freeland, WA, Washington, United States; 3 UConn Health, Farmington, Connecticut, United States; 4 University of Connecticut School of Medicine, Farmington, Connecticut, United States

## Abstract

FallsTalk is a one-month evidence-based falls prevention (FP) program that focuses attention on causes of an individual’s falls and encourages new FP behaviors. We translated the program for family caregivers of persons with cognitive impairment or dementia (PwCID) to administer to the PwCID and examined the number of new FP behaviors (#newFPBs) reported during the intervention period by the Caregiver. Thirty-four Dyads (Caregiver+PwCID) were trained to conduct brief daily FP discussions together using paper (FTCGnoTech) or computerized (FTCGTech, n=20) guidance. Dyads had FallsTalk training, daily discussions and weekly check-in calls. FTCGTech included discussion suggestions tailored to Caregiver concerns. To examine the use of technology, Poisson regression models compared #newFPBs between FTCGnoTech and FTCGTech and included covariates age (<80 or >=80), Mini-Mental Status Exam (MMSE >25 or <=25) and interactions. To evaluate the influence of Caregiver participation, #newFPBs reported by 115 non-demented clinical trial participants (no Caregiver- FT-ClTrNoCG) were compared with the Caregiver outcomes. Dyads using technology reported significantly more #newFPBs (MeanFTCGnoTech=5.34(SEM=0.68), MeanFTCGTech=8.46(SEM=0.76); p=.004) during the intervention month. A significant interaction was observed whereby Dyads with MMSE<=25 using technology, reported significantly more #newFPBs than the non-technology group (MeanFTCGnoTech=4.23(SEM=0.72), MeanFTCGTech=9.01(SEM=0.90); p=.047). Caregiver (n=34) involvement substantially increased #newFPBs (MeanFT-ClTrNoCG=1.39(SEM=0.15), MeanCaregiver=7.21(SEM=0.49); p<.0001), independent of technology. Across studies, participants or Caregivers for those with MMSE<=25 and <80yo reported significantly more #newFMBs (Mean=4.38(SEM=0.55) than those 80+yo (Mean=2.06(SEM=0.30); p=.0026). FallsTalk Caregiver provides an effective means to promote new Dyad FP strategies. The influence of Caregiver involvement and technology show promise in encouraging behavioral change to prevent falls.

